# Sources of Resistance to Common Bacterial Blight and Charcoal Rot Disease for the Production of Mesoamerican Common Beans in the Southern United States

**DOI:** 10.3390/plants10050998

**Published:** 2021-05-17

**Authors:** Daniel Ambachew, Jacqueline Joshua, Margaret T. Mmbaga, Matthew W. Blair

**Affiliations:** 1 Department of Agricultural and Environmental Sciences, College of Agriculture, Tennessee State University, Nashville, TN 37209, USA; ddemissi@tnstate.edu (D.A.); jacqueline.joshua@bayer.com (J.J.); mmbaga@tnstate.edu (M.T.M.); 2 700 Chesterfield Parkway W., Chesterfield, MO 63017, USA

**Keywords:** broad-sense heritability, common bean races and market classes, disease resistance candidate genes, genome-wide association study, principal component analysis

## Abstract

The gene pool of Mesoamerican common beans (*Phaseolus vulgaris* L.) includes genotypes in the small-to-medium-size seeded dry beans, as well as some snap beans from hotter environments adapted to the Southeastern United States. However, the warm and humid climate of the Southeastern United States is conducive to diseases such as Common Bacterial Blight (CBB) and Charcoal Rot (CR). The pathogens for these two diseases can survive long periods in infested soil or on seeds and are difficult to control through pesticides. Hence, field-level resistance would be the best management strategy for these diseases. The goals of this study were (1) to evaluate field-level resistance from the various commercial classes and subgroups represented in the Mesoamerican gene pool as sources for breeding beans for the region and (2) to evaluate genome-wide marker × trait associations (GWAS) using genetic markers for the genotypes. A total of 300 genotypes from the Mesoamerican Diversity Panel (MDP) were evaluated for CBB and CR in field experiments for three years. CBB resistance was also tested with a field isolate in controlled greenhouse conditions. The analysis of variance revealed the presence of variability in the MDP for the evaluated traits. We also identified adapted common bean genotypes that could be used directly in Southeastern production or that could be good parents in breeding programs for CBB and CR resistance. The GWAS detected 14 significant Single-Nucleotide Polymorphism (SNP) markers associated with CBB resistance distributed on five chromosomes, namely Pv02, Pv04, Pv08, Pv10, and Pv11, but no loci for resistance to CR. A total of 89 candidate genes were identified in close vicinity (±100 kb) to the significant CBB markers, some of which could be directly or indirectly involved in plant defense to diseases. These results provide a basis to further understand the complex inheritance of CBB resistance in Mesoamerican common beans and show that this biotic stress is unrelated to CR resistance, which was evident during a drought period. Genotypes with good yield potential for the Southeastern U.S. growing conditions were found with resistant to infection by the two diseases, as well as adaptation to the hot and humid conditions punctuated by droughts found in this region.

## 1. Introduction

The common bean, *Phaseolus vulgaris* L., is an important species to agriculture in the United States (U.S.) primarily produced as a commodity grain crop [1] and as a vegetable crop, known as snap beans [2,3,4]. Dry beans are valued at one billion dollars [5] and snap beans valued at 0.3 billion dollars [6] with a significant impact on the American economy. 

Dry beans are grown across a northern arc of U.S. states and a tier of Canadian provinces starting in Maine and spreading through New York, Ontario, Alberta, Michigan, going west all the way to the central valley of California.Due to climate change, plant diseases, and a loss of markets, the eastern and middle parts of this arc of U.S. states have become less productive, and production has moved westward to areas with irrigation in western Nebraska, the front range of the Rockies in Colorado, or the Snake River valley in Idaho, but more importantly, northward to North Dakota and southern Saskatchewan [7]. Seed production for dry beans is concentrated in Idaho. 

Snap beans, on the other hand, follow a seasonal worker and machine harvester migration from the Southern U.S., with the earliest and highest value fresh crops in Florida, Georgia, the Carolinas, and Tennessee, moving north to facilities that cut and freeze snap beans in Wisconsin at the height of summer [8].

The U.S. southeastern states have long growing seasons and have the capacity to grow the common bean at least twice a year based on spring and summer plantings. Dry beans used to be a common staple of the Southern diet together with corn but have waned given urbanization. Mixed crop vegetable farms, smaller organic farms, as well as backyard gardeners continue to produce both dry and snap beans in the region. Early production, local consumption, and proximity to markets favor larger scale snap bean production especially on the Cumberland Plateau of Tennessee [9]. 

Due to the hot climate, small-seeded Mesoamerican common beans do better in the Southeastern USA than large-seeded Andean gene pool beans. Other zones of production for dry beans with high day temperatures are found in the Central Valley of California; however, Southeastern production of the common bean has the most severe heat stress for the crop given its timing of production and high night temperatures with a range of warm-season diseases as described in Harveson et al. 2015 [10] and shown below. In addition, the summers of the Southeast are notoriously humid, affecting the proliferation of fungal and bacterial diseases.

Warm-season diseases of common beans include Common Bacterial Blight (CBB) caused by the pathogen *Xanthomonas axonopodis* (syn. *campestris*), and root rot caused by the soil fungal pathogens *Fusarium* spp. and the stem and root infecting *Macrophomina phaseolicola* (Mp). Among bacterial diseases of the common bean, CBB is the most important by far, as it is seed borne, remains in soil, spreads rapidly from leaf to leaf, and is found across a large range of environments [11,12,13,14]. Classification of CBB strains separates two pathovars: *X. axonopodis* var. *phaseoli* (*Xap*) and *X. axonopodis* var. *fuscans* (*Xaf*) [15]. 

*Macrophomina* is a genus of fungi especially important in areas where heat stress is followed by dry weather [16,17]. The disease that Mp causes is called Charcoal Rot (CR) of beans and is very destructive because the fungus strangles the stems at the base of fully-grown plants and even those approaching maturity, preventing water uptake or translocation from the roots and causing the whole canopy and pods with seeds to wither and dry. The result of CR is much smaller seeds being produced and much lower yields. 

While total crop failure has been attributed to CR on infested soil, the losses to CBB can vary greatly depending on humidity levels and inoculum levels. CBB often causes up to a 60% yield loss in susceptible common beans during favorable conditions for the breakout of the disease [14,18,19]. While pathogen races have been well studied in other common bean diseases, CBB and CR diseases have just recently had some analysis and development of differentials [20,21,22] for the naming of races and classification of varieties. 

Genetic resistance is the most effective strategy to control diseases in the common bean. The use of resistance (R) genes is environmentally friendly as they reduce the use of chemicals, especially fungicides, which can be harmful to humans, phytotoxic, or long lasting [23]. In the case of bacterial diseases, few chemical-control techniques are feasible. 

Despite the importance of genetic resistance, almost no R genes for these warm-season diseases have been cloned. There has been some success in breeding CBB-resistant lines such as VAX3, VAX4, VAX5, VAX6 [24], and USDK-CBB20 [25]. Registered varietal releases for CBB resistance include Coyne [26], Badillo [27], Lighthouse [28], Cayenne [29], and Panhandle Pride [30]. Studies on the inheritance of resistance to CBB have been initiated especially in the case of *Xap*, although less is known about *Xaf*. 

Recent reviews and reports listed 25 genetic factors or Quantitative Trait Loci (QTL) for CBB [14,18,19,24,31,32,33,34,35]. These include a major QTL on chromosome Pv08 (tagged with the marker SU91) and minor genes on chromosomes Pv07 (near the phaseolin or *Phs* locus) and Pv10, reducing leaf lesion size [19,23]. The QTLs on linkage groups Pv07 and Pv10 were associated with markers BC420 and SAP6 used in breeding [32]. Zapata et al. reported a dominant gene for CBB resistance that co-segregated with SAP6 [36].

Meanwhile, the study of resistance to CR disease started when Pastor-Corrales [37] evaluated 53 common bean genotypes for their reaction to *M. phaseolina* infection under field and greenhouse conditions, reporting only BAT477 and 14 other genotypes as resistant. Olaya et al. [38] studied the inheritance of resistance to CR and discovered two dominant complementary genes, namely *Mp1* and *Mp2* for resistance in the BAT 477 genotype, followed by Miklas et al., who discovered three QTLs in the DOR364 × Xan176 populations [39]. Hernández-Delgado et al. also reported that BAT477 resistance was controlled by recessive epistatic genes and a QTL on Pv01 [40].

Genome-Wide Association Studies (GWASs) mean the evaluation of marker × trait associations useful to QTL discovery in structured or unstructured populations rather than segregants from single crosses [41]. GWASs have been useful for the identification of candidate genes responsible for the expression of traits in a diverse group of genotypes for various crops. The current advancement in genetic markers has reduced the cost of genotyping allowing many marker × trait associations to be evaluated with loci covering a large part of the genome of the target organism. In recent years, GWASs have been conducted on a per-gene pool or combined basis for CBB disease in Andean and Mesoamerican beans [18]; as well as agronomic traits in Andean [42] and Mesoamerican [43] beans. For other diseases in beans, GWASs have been useful for anthracnose in the Andean gene pool [44], anthracnose and angular leaf spot diseases in Brazilian germplasm [45], *Rhizoctonia solani* in both Andean and Mesoamericans [46], and cyst nematode resistance in snap beans [47].

The goals of this study were (1) to evaluate field-level resistance from the various commercial classes and subgroups represented in a Mesoamerican Diversity Panel (MDP) as sources for breeding beans for the Southeast region of the USA and (2) to evaluate marker × trait associations using previously developed genetic markers for the genotypes and GWAS screening for two diseases, CBB and CR, which are prevalent in warm climates. Another goal of this project was to assess which common bean races, commercial classes, and genotypes adapted best to the region’s growing conditions or could be used as donor parents for breeding of locally adapted dry beans or snap bean germplasm. The MDP was evaluated over three years in a diseased plot to find field levels of resistance to CBB and CR with greenhouse validation of a subset of the GWAS panel with a field isolate of *Xap* to complement the field study. Seed yield data were collected as a measure of resistance to the diseases.

## 2. Results

### 2.1. Phenotypic Differences in the Mesoamerican Diversity Panel

**Figure 1:**
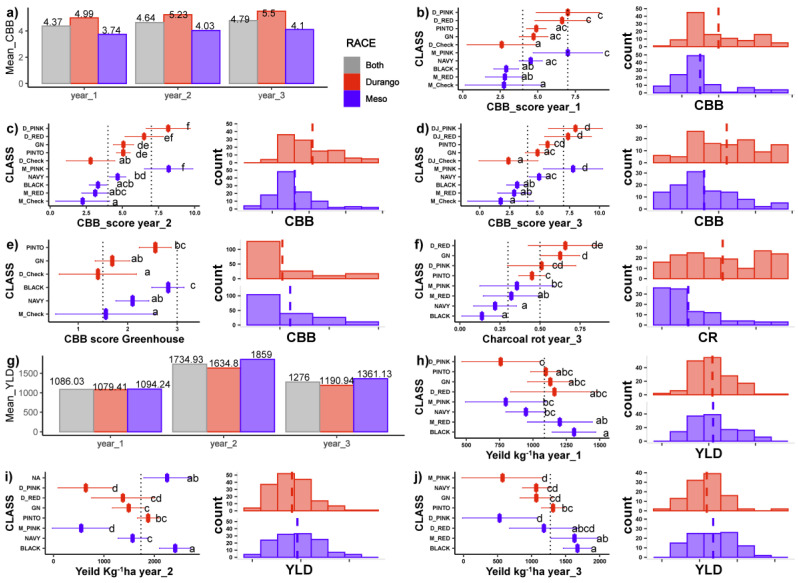
Performance of the Mesoamerican diversity panel common beans tested for Common Bacterial Blight (CBB) disease across years (**a**) or within each of Year_1 (**b**), Year_2 (**c**), and Year_3 (**d**) in the field site. Red color coding in the bar charts and distribution charts indicates race Durango, while blue color indicates race Mesoamerica. Gray color in bar chart is for overall averages. Further graphs show performance when inoculated with a CBB isolate in a greenhouse (**e**), the incidence of Charcoal Rot (CR) in the Year_3 field trial (**f**), and yield performance across (**g**) and within all Year_1 (**h)**, Year_2 (**i**), and Year_3 (**j**). Means and least squares means recorded on different market classes of the Durango and Mesoamerica races shown for each horizontal bar graph with error bars indicating 95% confidence intervals. Classes sharing the same letter were not significantly different (at *p* < 0.05, Tukey-adjusted). Classes that were identified as resistant (1 to 3 CBB score), moderately resistant (4 to 6), and susceptible (7 to 9) are separated by the vertical lines. Means for each race indicated in count distributions to the right of the horizontal bar graphs. The vertical lines on these histograms represent the means of the population.

The MDP showed a wide range of differences in seed yield. The highest mean seed yield (1723 kg ha^−1^) was obtained in Year_1 followed by Year_3 (1276 kg ha^−1^) and Year_1 (1086 kg ha^−1^). The MDP population mean and mean and mean seed yield productivity of races are indicated in Figure 1. In all the years, race Mesoamerica produced more than race Durango. The analysis of variance result for the incomplete block design of the Year_3 field experiment (Table 1) indicated the presence of significant differences within all the entries, test genotypes, between controls, and test genotypes against controls for the two diseases. The mean performance of the MDP against CBB was better in the first year as supported by lower values of the mean disease score. The bar plot and histograms in Figure 1 show that the mean CBB score in all field experiments for the race Mesoamerica was smaller than the race Durango, which was also true for CR disease. 

The minimum and maximum values of the CBB score for the MDP over the years ranged from one to two, which were the extremes of the scale used for determining resistance/susceptibility. Similarly, the full range of 0% and 100% CR incidence was recorded on the genotypes, with 12% showing no CR disease in the plot and 8% showing all plants infected (data not shown).

### 2.2. Correlation between Seasons and Climate Condition

**Figure 2:**
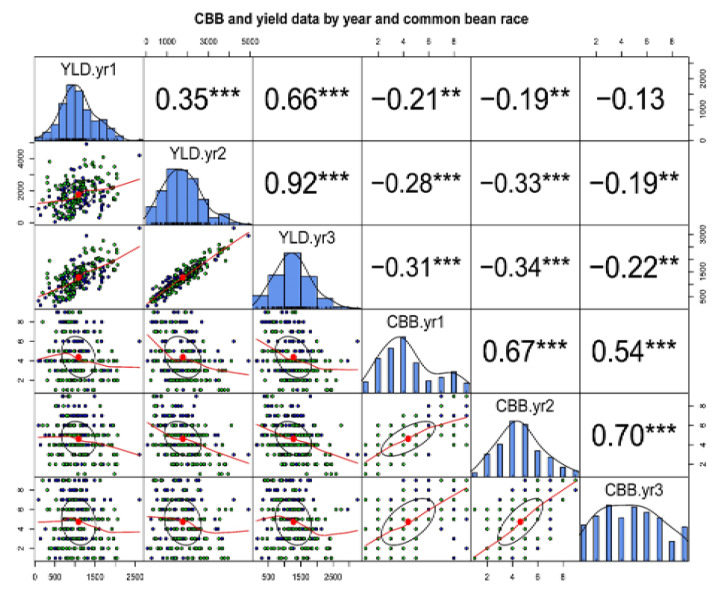
Pearson correlation between seed yield in kg/ha (scale shown on the second row and column) and CBB and CR disease scores (scale shown on the fourth and sixth row and column) for the Mesoamerican diversity panel grown over three years at the same site in Tennessee, with r-values (shown above the diagonal) and bivariate scatter plots (shown below the diagonal). Histograms showing the distributions for each trait found in boxes along the diagonal, with the abbreviation of the trait and year. Significance values for correlations indicated by asterisk ** *p* ≤ 0.01, *** *p* ≤ 0.001. Non-significance shown by the absence of asterisks.

The correlations between seed yields of genotypes and their reaction to CBB and CR in each year is indicated in Figure 2. Seed yield was highly correlated between years in a significant and positive manner. The highest magnitude of correlation was observed between the seed yield produced in Year_2 and Year_3 (r = 0.93, *p* < 0.001) followed by the correlation between the seed yield produced during Year_1 and Year_3 (r = 0.66, *p* < 0.001). The correlation between CBB disease scores in each year was also high and significant, with the highest level being between Year_2 and Year_3 (r = 0.57, *p* < 0.001). Seed yield and CBB showed highly significant and negative correlations, but of smaller magnitude. The correlation of years for different traits was affected by the prevailing weather conditions during the growing seasons of the three years.

The minimum and maximum daily temperatures and daily precipitation recorded during the three years’ growing seasons were recorded (Supplemental Figure S1). The maximum total precipitation (593 mm) was recorded during Year 1 followed by 489 mm in Year_2. Year_3 received the lowest total precipitation (484 mm). The minimum and maximum temperatures were higher in Year_3 followed by Year_2. 

Differences were partly due to planting times. The Year_1 growing season (April to August) was cooler with a higher amount of rain recorded, which affected the yield potential of the MDP beans in Tennessee. The Year_2 season (May to September) was conducive to CBB and CR diseases in the MDP as it was warmer. Rainfall was adequate, which helped the MDP produce a high yield even under higher CBB infestation. The Year_3 season (June to September) was much hotter and drier, which helped the buildup of CBB and CR diseases coupled with heat and water stress. Though the disease pressure was high, the MDP produced a better yield in Year_3 than in Year_1.

### 2.3. Differential Response among Bean Races and Commercial Classes for Disease Reaction

The performance of common bean genotypes against natural infestation of CBB and CR was assessed between two common bean races (Mesoamerica and Durango) and six market classes (small red, pink, pinto, navy, GN, and black beans; the two common bean races share pink and small red market classes). 

Out of 25 pink genotypes, thirteen were from Durango and twelve from Mesoamerica. Similarly, small reds were divided between the two races: 13 genotypes from race Durango and 21 genotypes from Mesoamerica. The analysis of variance of each field experiment (Years_1, _2, and _3) (Table 2) showed the presence of a significant difference among the market classes in terms of their reaction against CBB and CR. No significant difference was observed between the Mesoamerica and Durango races for CBB.

The error bars in Figure 1 indicate the upper- and lower-class boundaries showing the variability of each tested group over the years. The pink beans and the CBB resistant checks from both races were highly variable in their reaction to CBB over the years. The Mesoamerica race had better resistance against CBB than the race Durango. Black beans and small red beans are Mesoamerica beans that displayed higher resistance to CBB. The GN and navy beans showed moderate resistance. 

Similarly, small red, and pinto beans from the race Durango were also moderately resistant to CBB. Pink beans regardless of their race were susceptible to CBB. The mean performance for CR showed that out of all market classes, the Mesoamerica race was much better against CR than the counterparts of the Durango race.

Black and navy beans were resistant to CR with mean CR incidence below 0.3. The two common bean races made a small contribution to the total variation observed for CBB reactions in Year_1 and Year_3 and relatively higher contribution (21.36%) in Year_2. The difference in races contributed 30.8% of the total observed variation for CR reaction (Table 2). Out of the total variations observed in the populations evaluated for CBB reaction, about 25.6% was in Year_1, 27.95% in Year_2, and 30.41% in Year_3. Similarly, market classes contributed 7.6% of the variation for CR in Year_3 (Table 2). 

The mean performance of genotypes against CBB was also tested using data from the three field experiments for combined analysis. The effect of individual genotypes was tested by fitting the GLM considering common bean races and market classes as fixed effects and individual genotypes as random effects. Genotypes were tested using the model error mean square, and the effect of races and classes were tested using the genotype mean square as the error term in the GLM. The global analysis for CBB also showed the presence of significant difference between seasons, classes, and individual genotypes (Table 3). The CR disease resistance data were taken only in one year when the incidence of the pathogen was high, and therefore, they could not be subjected to a combined analysis.

The MDP was also assessed for CBB and CR dual resistance using a scattergram by plotting CBB vs. CR on the x and y axis, respectively. The scattergram (Figure S1) identified six groups of common beans based on their reaction to CBB and CR diseases. The first group consisted of genotypes resistant to both diseases in this group, black and small red beans from the race Mesoamerica and GN beans from the race Durango being dominant. The second group consisted of genotypes resistant to CBB, but susceptible to CR, this group being dominated by the GN and pinto beans from the race Durango. The third group was moderately resistant to CBB, but susceptible to CR. The fourth group dominated by the GN, pinto, small red, and pink beans from the Durango race were susceptible to both diseases. The fifth group was comprised of pinto beans, which were susceptible to CBB and resistant to CR. Group 6 was dominated by black and pinto beans and consisted of genotypes that were moderately resistant to both CBB and CR. Four genotypes, GN#1Sel27, IBC-3, Xan176, and ND021717, were among those that showed high resistance to both CBB and CR. These genotypes were also among top 5% high-yielding genotypes (Table S3) and could be sources of combined resistance to CBB and CR diseases.

### 2.4. Mean Yield Performance of Commercial Classes

The yield potential of MDP under the pressures of CBB and CR was also evaluated. The analysis of variance of each field experiment showed the presence of significant difference among the market classes for yield. Like the diseases, no significant difference was observed between the Mesoamerica and Durango races for yield (Table 2). Races made no contribution to the total variation, while market classes contributed 12.69% in 2014, 29.19% in Year_2, and 30.66% in Year_3 of the total variation observed on seed yield. The global analysis for seed yields also showed the presence of significant differences between individual genotypes (Table 3). More than one third (35.8%) of the total variation observed in the global analysis was contributed by the individual genotypes. The effect of the difference between seasons on the performance of genotypes was also significant: it accounted for 21.1% of the observed variation.

### 2.5. Greenhouse Validation of CBB Results

We found a significant difference among individual genotypes and market classes evaluated against CBB in a greenhouse experiment (Table 3). From the total observed variation, market class subgroups and individual genotypes contributed 17.82% and 32.18%, respectively. Similarly, the global analysis from the three field experiments confirmed the presence of significant variation among individual genotypes, as well as among market classes (Table 3). Individual genotypes contributed 41.34% of the total variation against CBB, followed by bean races (13.23%). The global analysis also showed that the difference in the years had a smaller contribution (0.42%) to the total observed variation against CBB, and eleven-point-six percent of the variation was contributed by market classes. Individual genotypes and market class subgroups also showed significant differences in their seed yield productivity.

### 2.6. Orthogonal Contrasts and Determination of Best Genotypes

We used one-degree-of-freedom contrast on the combined data to compare the means of different market classes within and between races (Table S2). These contrasts included means of races compared to CBB = resistant checks. Means of different market classes were also compared. In those comparisons, the GN and pinto beans from the Durango race and black and small red beans from the Mesoamerican race were better in CBB resistance than other market classes. No significant difference was observed between the white beans (the GN and navy beans) in their reaction to CBB. The Mesoamerican red beans were more resistant to CBB as compared to the red beans in the Durango race.

Generally, the result indicated that Mesoamerican beans were more resistant to CBB as compared to their Durango counterparts. The mean separation from the combined data from the Year_1 to Year_3 growing seasons identified CBB-resistant and high-yielding genotypes in Tennessee growing conditions. With the selection of the top 13% best performing genotypes against CBB (Table S4), a total of 29 genotypes were identified as resistant to CBB with a mean CBB score of < 2.67 + 0.72. All the CBB resistant checks were included in this selection. Similarly, eleven genotypes were identified among the top 5% highest yielding genotypes with a mean production of ≥2161 + 278 kg·ha^−1^. Genotypes IBC-3, PR0443-151, Dehoro, and ND021717 were resistant to CBB with higher mean seed yield productivity. Out of the CBB resistant checks, the best yielder was IBC-3.

### 2.7. Principal Component and Phylogenetic Tree Analysis

**Figure 3:**
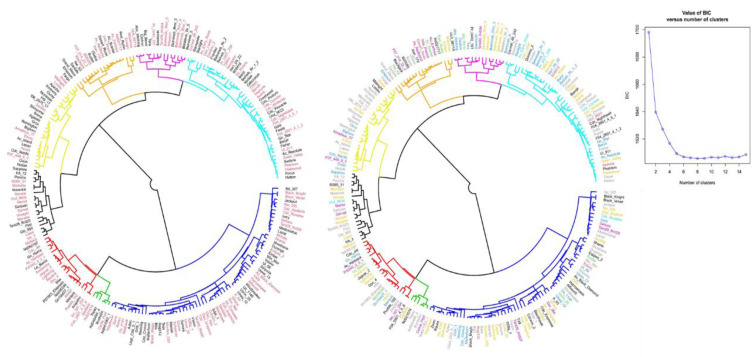
Phylogenetic relationship of genotypes from different market classes in the Mesoamerica and Durango races. The different colors of the branches represent the new clustering resulting from the K-means hierarchical clustering of genotypes. Text colors represent the two common bean races (left) and eight market classes (right).

After removing SNP markers in strong LD (r^2^ > 2) and maintaining only SNP loci with MAF above 0.05, a dataset of 2605 markers was used to perform PCA and phylogenetic tree analysis. The first two principal components accounted for 28% of the total explained variation (Figure S1), however with no clear separation between races and market classes. Phylogeny found only two major groups (Figure 3) with five and three dendrogram clusters found by the K-means method. The identified clusters were composed of genotypes from different market classes and races indicating the presence of genotypes that were more related to genotypes in a different market class or race and distant from genotypes of their own market class and race. 

### 2.8. Genome-Wide Association Analysis Result and Associated Genes

**Figure 4:**
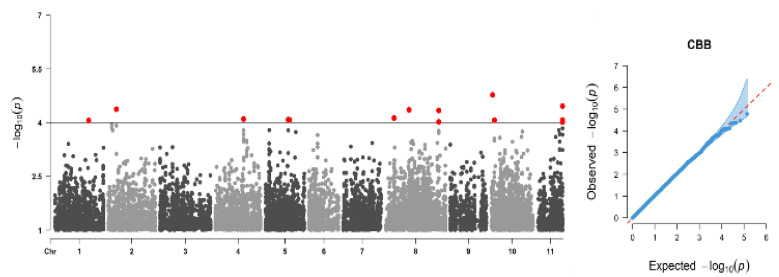
Manhattan (left) and QQ (right) plots of CBB associations; SNPs are ordered by physical position and grouped by chromosome. The black dashed line indicates the genome-wide significance threshold. The SNPs highlighted in red had *p*-values lower than the exploratory significance cutoff value = 1 × 10^−4^ (LOD = 4).

A total of 136,506 SNPs and 220 genotypes were retained after filtering out SNPs with MAF < 0.05, and this data set was used in the GWAS for CBB. CBB had a broad-sense heritability of 0.53, which was suitable to consider the trait for GWAS analysis. GWAS was visualized in Manhattan plots (Figure 4) and identified 14 significant SNPs residing on six chromosomes (Pv02, Pv04, Pv05, Pv08, Pv010, and Pv11) at a *p* =1 × 10^−4^ significance cutoff (Table 4). No significant association was detected at the *p* = 1 × 10^−6^ threshold level based on Bonferroni correction for an independent number of tests. However, using the exploratory significance level of LOD = 4, strong associations were found such as with S10 1437174 on chromosome Pv10. A total of 89 genes were annotated around the significant SNPs located within the 100 kb window up- or down-stream of the significant SNPs. Most of the genes were located on two chromosomes: Pv 10 (40 genes) and Pv 08 (35 genes). The name, direction, position, and function of the genes are presented in Table S5. The effect of SNPs and their contribution to phenotypic variations were very minimal. The total variation explained by all 14 significant SNPs was 41.7% (Table 4). Many of the SNPs detected produced multiple hits when aligned to the G19833 genome, making the identification of the right candidate genes complex and difficult. Nevertheless, we were able to identify candidate genes that were involved in the disease defense mechanism, and the most important genes associated with disease resistance were discussed.

## 3. Discussion

Dissecting the genetics of inheritance for quantitative traits is an important aspect of plant breeding in general and in bean breeding. The GWAS was a helpful tool with a better power in this regard, with a better resolution than QTL mapping. In our study, we used a high LOD score and found significant SNPs despite the high-level cutoff value of the Bonferroni test using the total number of tests or using the number of independent tests estimated using SimpleM methods. This indicates that the inheritance of CBB resistance is quantitative and environmentally sensitive, making the discovery of small effect markers associated with CBB-resistant genes difficult.

The interaction of the resistance gene/QTL with the environment is important in the case of CBB where some variability for the pathogen has been recorded [13]. Having this in mind, we used exploratory significance cutoff values. When we reduced the significance cutoff level to *p* < 1 × 10^−4^, we found significant SNPs associated with many genes involved in plant defense to diseases. This indicated that the two significance cutoff methods were more stringent to the extent they could not detect any of the associated SNPs.

This might be because the phenotypic variation explained by each significant SNP being low, ranging between 2.8% and 3.4% of the observed phenotypic variation to CBB. Most of the identified genes in this study resided on two chromosomes: Pv08 and Pv10.

A review of QTL studies conducted on the inheritance of CBB resistance in common beans reported that QTLs conferring CBB resistance were distributed across the genome, [19] and more than 24 major and minor effect QTL have been reported [14,24,35], the most popular scar markers SU91 and SAP6 being located on Pv08 and Pv10, respectively (see the reviews by Singh and Schwartz [14] and Singh and Miklas [24]. BC420 was also reported on chromosome Pv06 [19,34]. Very recently, Zhu et al. [19] identified two new codominant markers, BMp10s174 and BMp10s244, on Pv10 where SAP6 was found in between these two markers. In support of our study result, quite a number of candidate genes associated with CBB resistance were discovered on Pv10 by Zhu et al. [19].

The highly significant SNP S10 1437174 was close to a cluster of 18 genes, many of them involved in plant defense mechanisms. Among these, *PvUI111.10G005900* was located 89kb downstream, encoding a zinc finger FYVE containing proteins (GDSL esterase/LIPASE LTL1) involved in plant disease resistance and stress responses [48,49] and *PvUI111.10G006100* positioned 70 kb downstream encoding the PAR1 protein that promotes the activation of defense responses during disease infection [50,51,52].

Another significant SNP on chromosome Pv10 was S10 3398159. We found 22 genes surrounding it. Three of these genes *PvUI111.10G019000*, *PvUI111.10G019400*, and *PvUI111.10G019700*, were found 71 kb, 37 kb, and 20 kb downstream, respectively, of the significant SNP. The first encoded an alkane hydroxylase involved in the lipid signaling response to pathogens, which is essential for plant resistance to pathogens [53]; the others were unrelated genes. *PvUI111.10G020700* was further from the SNP (73 kb downstream), but encoded a SKP1-like proteins, which is known to regulate the abundance of disease susceptibility factor RACB [54].

Four significant SNPs associated with 35 genes were found at *Pv*08. Among these genes, *PvUI111.08G226000* resided 50kb upstream of S08 55730740 encoding the subtilase protein family composed of peptidase S8 and the protein-associated domain (PA). This protein family is involved in plant defense responses against most diverse pathogens [55] via pathogen recognition and immune priming by programmed cell death [56]. Ten genes encoded the proprotein convertase subtilisin/KEXIN protein family. These proteins are believed to be involved in the activation of other proteins involved in plant defense to diseases.

Another important gene was *PvUI111.04G094600* found at *Pv*04 70kb upstream of the significant SNP S04 29756136. It encodes small zinc finger proteins that play an important role in the regulation of plant responses to biotic stresses, plant growth, and development [57,58]. The rest of the genes encoded proteins either involved in the activation of growth and cell division or acted as transcription factors. 

Identification of common bean genotypes with good environmental adaptation and disease resistance involves screening and selecting for reaction to diseases while simultaneously evaluating for higher seed yield and often aerial and root biomass needed for productivity. In this study, we evaluated the adaptation of 300 commercial common bean genotypes to middle Tennessee conditions and screened them for disease resistance against the natural field infestation of CBB and CR diseases and artificial greenhouse inoculation of *Xap*.

The frequent rainfall, combined with warmer temperatures during the growing seasons, helps the buildup of CBB. The weather conditions during the Year_3 growing season were relatively hot, and the rainfall received was smaller than the other two years, which led to the buildup of CR disease. 

Hot, dry weather promotes the infection and development of CR [16,17]. The large positive correlation of CBB reaction and seed yield between the years indicated the similarity of weather conditions over the years (Figure 3). These conditions facilitate the screening of genotypes under natural field infestations.

The analysis of variance indicated the presence of significant variation among common bean genotypes and market classes considered for the study, in seed yield potential and reaction to the two diseases, CBB and CR. The lack of significant differences between the two common bean races (Mesoamerican and Durango) over the years indicated that they were similar in terms of their reaction to CBB and adaptability to environments in middle Tennessee. This was also supported by the results from the PCA and the phylogenetic analysis done on the SNP data of the panel. 

Indeed, the clustering of the genotypes based on PCA and the phylogram from the genotypic and phenotypic data showed each cluster was a mixture of genotypes from different groups (races Mesoamerica or Durango). This might be because the genotypes shared a common genetic background from the two races because of the inter-racial hybridization or effects of some inter-specific and frequent inter-gene pool hybridizations that occur for common beans of the Mesoamerican gene pool. 

A review by Singh and Miklas [24] reported sources of resistance to CBB from primary, secondary, and tertiary gene pools, which are sources of the four popular QTLs associated with CBB resistance. These QTLs have been used to augment CBB resistance into commercial bean varieties and breeding lines through marker-assisted selection. For example, the great northern common bean landrace Montana No. 5 [12,18,59,60,61] and inter-specific breeding lines XAN and VAX were used to develop CBB resistance [24,32,34]. Notably, tepary beans (*Phaseolus acutifolius*, Gray) lines PI319443 and PI440795 have been used to develop CBB-resistant genotypes by most of the breeding programs in North America [18,23]. GN#1Sel27 was developed from Montana No. 5 and tepary bean parents [62], which carried SAP6 and BC409 SCAR markers [63], and it was used as a CBB resistance source to develop various bean breeding lines and a black bean genotype, IBC-3 [64] IBC-6 (small red), IBC-8 (small white), and IBC-10 (small black). CBB-resistant genotypes were developed from the same inter-specific cross between *P. vulgaris* and *P. coccineus* [64], but have a different market class. Likewise, different inter-specific and inter-racial hybridizations were implemented to develop climate-resilient, high-yielding genotypes [65]. However, genotypes under different market classes showed a significant difference in their response to CBB and CR. This suggests that some sort of variability existed in market classes and individual genotypes.

As practical results from this study, we revealed that Mesoamerican red and black beans resisted CBB better than the other market classes. Generally, pink beans were susceptible to CBB. Disease resistance may not always correlate with high yield, and this study showed that only four genotypes combined CBB resistance with yield. The rest of the resistant varieties including the CBB checks produced lower yields than the top 5% of genotypes that had a better yield (Table S3). Among the CBB-resistant checks, Coyne was a great northern bean genotype developed from a three-way cross and released for its CBB resistance [26]. ABC-Weihing is a registered genotype, developed for its multiple resistance to rust and Bean Common Mosaic Virus (BCMV) and enhanced resistance to CBB. The genotype carries the SU91 marker to CBB resistance [66]. GN#1Sel27 has been widely used as a CBB-resistant parent in most of the CBB resistance breeding programs in the world and transmits the SAP6 marker [13,26]. OAC Rex was developed from interspecific hybridization of tepary and common beans and had a moderate resistance to CBB. The genotype is known to carry a major QTL linked to scar marker SU91 on chromosome Pv08 [23,24,32,67]. OAC Rex was used as a parent for many CBB-resistant breeding lines and the recently released genotype Lighthouse [28]. Finally, BelNeb-RR-1 was a great northern released germplasm resistant to CBB and halo bacterial blight diseases, which has also been used as a source of general bacterial resistance by many breeding programs [68,69]. 

The newly identified genotypes from this study in Tennessee that were useful for CBB resistance with high yields included ICB-3, ND021717, Dehoro, and PR0443-151. These genotypes were evaluated at least one generation during their development in a humid tropical environment where CBB and CR are present. ICB-3 was a small black bean genotype developed in Puerto Rico from the cross GN # 1 Sel 27 × PC-37′ and released there for its resistance to CBB and good yield potential [24,64]. It is also reported to have some resistance to CR. ND021717 is a CBB-resistant black bean obtained from the cross VAX-4/Raven/T-39 [70]. Most breeding lines with the ND code (from North Dakota State University), as well as lines from university-based breeding programs in the states of Michigan and Nebraska are planted for at least one generation in a winter nursery in Puerto Rico where CBB and CR are important diseases. ND021717 was used as a parent to develop “ND Twilight”. Dehoro is a small red bean with multiple disease resistance including BGYMV, BCMV, ALS, and rust; however, no CBB resistance was previously reported on this released variety until this study. Finally, PR0443-151was reported to have adaptation to low soil fertility and root rot resistance, but has never been shown to have CBB resistance before or the dominant I gene for BCMV resistance. 

Multiple resistance to diseases is common in common beans. Several genotypes and breeding lines have been developed for multiple disease resistance. Our study revealed the presence of combined resistance to both CBB and charcoal rot diseases. Pastor-Corrales and Abawi [37] reported resistance to charcoal rot in common beans. XAN176 is a black breeding line developed for CBB resistance and has moderate resistance to charcoal rot [22,39,40]. Other Mesoamerican beans like BAT477 [22,38,40], SEQ 12, N.8025, TLP 19, and G4523 were considered resistant differentials to screen for charcoal rot resistance [64]. Several genotypes and breeding lines have been developed for multiple disease resistance. 

Overall, our study showed the presence of dual resistance for CBB and CR diseases, which could be exploited in the breeding program aimed to develop common bean (dry or snap) varieties for hotter environments like Tennessee. Given their background information and their performance in this study, XAN176 and IBC-3 can be a good resistance source of dual resistance and used as a parent to improve snap beans. Similarly, Coyne, GN#1sel27, OAC Rex, and BelNeb-RR-1 are among the good sources of CBB resistance to consider. In genomic results, our study identified candidate genes controlling CBB resistance in common beans. This information can be used to further understand how these genes function in plant disease defense through RNA and differential expression analyses and how they can be employed in crop improvement through marker-assisted selection or genome editing and gene engineering. 

## 4. Materials and Methods

### 4.1. Experimental Site Description

The study was conducted in Nashville, at the Tennessee State University (TSU) Agricultural Research and Extension Center (AREC) in the southeastern United States of America (USA). The research station is located at 36°10′36″ N and 86°49′34″ W with an altitude of 128 m above sea level and is in the floodplain of the Cumberland River. The field experiments were conducted on a soil type that is silt loam and is of the Byler series. Annual rainfall at the site is approximately 1200 mm per year with summer months falling off from a high of 150 mm, but varies in intensity, number of rain events, and amounts per event depending on tornado fronts (spring), cool fronts (summer), and hurricane season (autumn).

Monthly average temperatures during the summer months are 30 °C to 32 °C with a drier period in August and September. The annual mean daily temperature is 15 °C, but summer maximums are near 40 °C day and 30 °C night temperatures. The Köppen climate classification is humid sub-tropical (Cfa). The periods of common bean production in Tennessee are in the late spring or early fall and flank the intensely hot summer growing season for other crops. 

The common bean is grown commercially as snap beans and as dry beans or vegetables in backyard gardens throughout the state. Nashville, being in middle Tennessee is representative of the state’s growing conditions. The time of planting varies with the geographical regions of the state. In the west and southern middle Tennessee, beans could be planted from April 15 to 20, in the highlands and Cumberland plateau from May 1 to August 1, and in upper east Tennessee from May 20 to July 20. To be representative of these various planting dates in our state, we planted our experiments at the AREC in mid-April for Year_1, mid-May for Year_2, and mid-June for Year_3. Each planting period was useful for different disease testing, as well as adaptation trials for common bean growth in the state. The experimental site was developed as a disease plot for CBB and CR diseases’ natural infestation with a repeated planting of “Top-crop”, a susceptible bush-type snap bean and local commercial genotype in spreader rows throughout the field. A Greenhouse (GH) trial was conducted in Year_2 at the same AREC station under a glass-roofed GH with metal tables. Potted plants in the GH were grown during February to April under natural light and were inoculated with a field isolate of the CBB pathogen *X. a.* cv. *phaseoli*, as described below.

### 4.2. Plant Materials

The common bean genotypes were from a Mesoamerican Diversity Panel (MDP) used for agronomic tests and described previously [43,46,71]. The panel included 300 genotypes, mainly commercial cultivars, as well as breeding lines of *P. vulgaris* from the Mesoamerican gene pool (Table S1). Seed was obtained from C. Urrea of the University of Nebraska, Scotts Bluff Station, which was part of a harvest of the USDA Common Bean Coordinated Agricultural Project (BEAN-CAP) assembled by multiple U.S. universities and institutions and sponsored by the United States Department of Agriculture/National Institute of Food and Agriculture (USDA-NIFA). The genotypes represented two groups about equally: from race Mesoamerica (with 135 entries) and from the Durango-Jalisco complex (with 165 entries), for simplicity referred to as race Durango. Furthermore, the genotypes represented six market classes: pinto (with 99 entries), small red (34), great northern (44), navy beans (54), pink (25), and black beans (44). Eight CBB-resistant checks were selected including GNStar, GN#1Sel27 [12], Coyne [26], ABC-Weihing [66], and Weighing [72] from race Durango and IBC-3 [64], OAC Rex [23,73], and XAN176 [39] from race Mesoamerica. Information on the resistance or susceptibility status to CR was not available for the checks, but we were able to find quantitative differences between races and classes among experimental genotypes for this disease as well. Differences for CBB were also evident for the same genotypes.

### 4.3. Field Trial Management and Design

The experiment of 300 genotypes was first planted in the field and evaluated for a natural infestation of CBB using a single replication during Year 1 and Year 1 based on seed availability. In these trials, genotypes were arranged in continuous rows of 2 m in length, leaving 40 cm between rows, each row representing each genotype, and individual plants spaced 10 cm apart.

Once seed was multiplied, the third experiment was conducted in Year 3, when the whole set of genotypes was evaluated for CBB, CR, and yield using an augmented design [74] with ten incomplete blocks containing 30 genotypes and four controls (GNStar, GN#1Sel27, IBC-3, and OAC Rex) per block. The genotypes were arranged in plots consisting of two rows with the same length and planting distances used previously (2 m long, 40 cm between rows, 10 cm within rows).

While genotypes were not replicated, the controls were randomized to each incomplete block, and the four checks were replicated ten times to estimate the error variance. The planting dates were 15th of April, 15th of May, and 17th of June in Year 1, Year 2, and Year 3, respectively, for the three years to represent different possible times using bean planting in the three regions of Tennessee.

### 4.4. Plant Trait Measurements

The disease reaction to CBB and CR pathogens was assessed by collecting data on the disease score, the disease incidence, and the effect on seed/grain yield. Both disease evaluations were based on the expert evaluation of typical symptoms guided by a plant pathologist and breeders with many years of combined experience in common beans. 

All disease reaction data were collected at mid-pod filling through the maturation stage of the plant development. CBB disease data recording was based on the CIAT disease evaluation practical guide [75]. 

The CBB lesion level of leaf damage was recorded using a score from 1 to 9, where 1 was assigned to low or no infection on leaves and 9 was assigned to severe infection with large halos around lesions and loss of leaf area through desiccation and leaf drop. Data on the incidence of CR was collected as a proportion of the number of infected plants to the total number of plants within a plot. 

At harvest, seed yield was recorded on a plot basis using a PRACTICUM1102-1S sensitive digital balance with a capacity of measuring up to 1500 g and a 0.01 g scale (Sartorius Weighing Technology GmbH, Gottingen, Germany). Finally, the plot yield was converted to kg ha^−1^ based on the plot size.

### 4.5. Greenhouse Evaluation with Field Strain

To confirm or refute the CBB reactions observed for the panel in the field, a subset of 120 genotypes was selected for GH evaluation. The genotypes included in this set represented four market classes (black, navy, GN, and pinto) with each market class represented by 30 randomly selected genotypes. In addition, seven known disease-resistant checks (AOC Rex, ICB-3, Coyne, ABC-Weihing, Weihing, GNStar, and GN#1Sel27) were grown in the experiment. 

For inoculation, a field isolate of *Xanthomonas axonopodis* pv. *phaseoli* (*Xap*) was collected from infected bean plants in the Year_1 experiment at the Tennessee State University AREC as described above, to use in winter Year_2 for GH inoculation. 

Species and pathovar confirmation for the bacterial strain was done by PCR primers specific to *Xap*, based on the conserved gene sequences calculated from the sequencing done in previous research done by Dr. David Studholme of Exeter University and his group [76]. Isolation, purification, and inoculum preparation of the bacterial pathogen were done following the procedure of a practical greenhouse testing guide from CIAT [75].

The CBB evaluation was conducted using a randomized complete block design (RCBD) experiment with three replications during the period of January 15th to the end of April based on the time needed to read the CBB reactions of each plant. 

Growth conditions of the GH grown plants were as follows: three repetitions of two seeds each of each genotype were planted in 3.75 L (one-gallon) pots with sterile Miracle-Gro potting mix (Scotts lawn products, Inc., Marysville, OH, USA). After germination, the best of the two plants was kept and the extraneous seedling removed. The now single plants in each pot were inoculated at 17 days after planting on the most recently expanding leaf using the syringe method and a liquid bacterial culture of the *Xap* field isolate. 

This liquid suspension was from a 24 h old culture of bacteria, which was grown to a concentration of 5 × 10^7^ colony forming units (cfu)/mL in Erlenmeyer flasks held at 26 °C. Leaves were approximately 70% expanded and were monitored for symptom development while being maintained at a controlled temperature of 26 °C ± 3 °C (day)/20 ± 3 °C (night) °C with the Relative Humidity (RH) between 70 and 80%.

Plants were fertilized using granular Miracle-Gro garden feeder (24-8-16 N-P-K) twice at the amount of ¼ teaspoon per pot during the growing season and before the taking of disease ratings. Watering of the plants was done overhead as needed with a sprinkler hose and watering wand.

Disease scoring was done for leaf symptoms, primarily lesion size and yellow halo, 15 days post-inoculation according to the 1 to 9 disease scale described above. The evaluation was made right at the appearance of the first of the inoculated symptoms rather than later or on older plants, where symptoms can be mixed up with other leaf damage.

### 4.6. Phenotypic Data Statistical Analysis

Histograms were drawn for each combination of trait and year showing the population distributions found for CBB or CR resistance and yield for the genotypes from the Mesoamerican Diversity Panel (MDP) as evaluated in Year 1, Year 2, and Year 3. Two-way Analyses of Variance (ANOVAs) were conducted on the weighted means of seed yield and disease resistance data from the field and greenhouse experiments using General Linear Models (GLMs) in each case.

The variance components were computed using the “nested” and “Varcomp” procedures in Statistical Analysis Software (SAS) v9.4 (SAS Institute ©2000–2012, Cary, NC, USA), considering bean races as groups and bean seed market classes as subgroups. The data for Year 1 and Year 2 were unbalanced, so we tested the groups by taking the mean square of classes as an error term and the mean square of the error from the GLMs to test subgroups. In all the analyses, we used a random effects GLM. The Year 3 field experiment data were analyzed using the “Macro-augments” subprogram in SAS from Prasad and Gupta [77]. This allowed us to generate all orthogonal contrast coefficients among test genotypes, controls, and test genotypes against controls. Standard errors of differences in the analysis of variance between two test genotypes appearing in the same block, two test genotypes not appearing in the same block, two controls, one test genotype, and one control were also calculated in the same manner.

In addition, to confirm the presence of genotypic differences in terms of plant performance, seed yield, and CBB disease resistance, we combined the data from the three years of field experiments (denoted as “global analysis” hereafter). The homogeneity of variances was evaluated using the Bartlett and Kolmogorov–Smirnov (K-S) tests. To compare the effect of years on the yield performance of genotypes and their reaction to CBB, we conducted Pearson correlation on the three years of field data. For the sake of comparison, we only used genotypes with three years of data. The correlation and density plots were visualized using the “pairs, panels” function of the “psych” Package in R [78]. The variance components were estimated using the “Varcomp” procedure in SAS. Finally, we compared the means of market classes in each year. Lsmeans with the grouping and error bars and histograms with population mean of races were plotted using the “ggplot2” [79] and “easyGgplot2” [80] packages in R. We also compared the means of each genotype from global field analysis and greenhouse data.

### 4.7. Genotypic Data Analysis, Genotype Relationships, and Principal Component Analysis

The Middle American bean Diversity Panel (MDP) had been genotyped previously via Genotyping-By-Sequencing (GBS) using libraries constructed for 452 genotypes. The data were generated, processed, and aligned against a Phytozome 2.1 reference genome for *Phaseolus vulgaris* deposited and available at https://phytozome.jgi.doe.gov/pz/portal.html#!info?alias=Org_Pvulgaris (accessed on 5 May 2020), as explained in Oladzad et al. [71]. The 194,457 associated SNPs including imputed HapMap were downloaded from the Feed the Future (FTF) and USDA common bean coordinated agricultural project (BEAN-CAP) website (http://www.arsftfbean.uprm.edu/beancap/research (accessed on 5 May 2020) hosted by the Agricultural Research Service. In addition, the unimputed HapMap data were from https://doi.org/10.25387/g3.7965305 (accessed on 5 May 2020) [71].

A phylogenetic tree was made in this study for the 220 MDP genotypes as a variable for the GWAS analysis of our phenotypic results. SNPs with an MAF lower than 5% and heterozygosity above 2% were removed using TASSEL software V.5.2 [81]. After filtering, the remaining SNPs were used for the genome-wide association study. Linkage Disequilibrium (LD) pruning with r^2^ < 0.2 was done using PLINK [82] software to obtain a set of unrelated SNPs to evaluate the phylogenetic relationship and principal component analysis.

The SNP data were then converted from HapMap to numerical format using GAPIT3 [83] software in R. From that dataset, a phylogenetic tree was constructed using Ward’s hierarchical clustering and agglomerative algorithm [84] on Nei’s genetic distance [85] calculated with the “Gdist” function of the “NAM” [86] R package. Other packages in R including “dplyr” [87] and “adegenet” [88], were used to process and analyze the SNP data. The number of clusters was determined based on the basic information criteria obtained from the “find.clusters” function in the “adegenet” package. Subsequently, the “dendextend” [89] and “circlize” [90] packages were used to plot the phylogram. A PCA was also carried out to determine sub-populations based on the LD-based pruned SNP dataset using “prcomp” function of the “stats” package [79].

### 4.8. Genome-Wide Association Study

GWAS analyses were performed with TASSEL software V.5.2 [81] using the Mixed linear Model (MLM). The first three Principal Components (PCs) calculated from the LD pruned dataset as described above were included as a covariate in the MLM model to control for population structure. Kinship was also calculated using the normalized IBS [91] plugin in the TASSEL software and included in the model in addition to the PCs when using MLM in TASSEL. 

Manhattan plots and QQ plots were generated using the “CMPlot” R package [92], and significance levels were established using a Bonferroni correction at *p* < 0.05 based on the effective number of independent tests. This was determined via SimpleM [93]. We also used an exploratory significance cutoff at *p* < 0.0001. When reporting significant SNPs from each GWAS analysis, the SNP with the lowest *p*-value was chosen to represent each locus of interest.

The significant SNPs were positioned to the *Phaseolus vulgaris* v1.0 reference genome (G19833) [94] using Jbrowse in Phtozome v.13 [95] to assess candidate genes in a ±100 kilobase (kb) window, positioning the significant SNP at the center. The literature was consulted to determine the function of the candidate genes found in the significant trait × marker association windows.

## Figures and Tables

**Table 1:** Significance of entries, test genotypes, controls, and test genotypes against controls for common bacterial blight and charcoal rot in 248 common bean genotypes evaluated in an augmented design experiment.

Source	df	CBB	CR	Seed Yield
Block	9	0.78	0.02	1744.29
Entries	211	6.64 **	0.11 **	310,800.47 **
Test genotypes	207	5.34 **	0.10 **	307,463.3 **
Controls	3	96.69 **	0.52 **	134,277.37 **
Test vs Controls	1	5.69 *	0.31	1,262,515.75 **
Error	27	0.83	0.05	819.96
Corrected Total	247			

df = degrees of freedom; asterisks indicate * Significant at 5%, ** significant at 1%.

**Table 2:** Analyses of variance for common bacterial blight (CBB) or charcoal rot (CR) resistances, and seed yield (kg ha^−1^) for Durango and Mesoamerica races and seed color commercial classes within the Mesoamerican diversity panel for common bean genotypes evaluated over three years in Tennessee, showing sources of variation, degrees of freedom (df), mean squares, error, variance components and percentages.

TRAIT	YEAR	Source of Variance (df)	Mean Square	Error	Variance Component	Percentage of Total Variance
Seed Yield (kg ha^−1^)	Year_1	RACE (1)	52,806	CLASS	−9960.07	0
		CLASS (8)	570,449 **	Error	24,191	12.69
		Error (201)	166,757		166,757	87.33
	Year_2	RACE (1)	5,097,230.7	CLASS	−50,466	0
		CLASS (8)	4,840,309.78 **	Error	253,244	29.19
		Error (201)	614,247.5		614,248	70.81
	Year_3	RACE (1)	1,545,948.98	CLASS	−26,236	0
		CLASS (8)	1,994,374.83 **	Error	105,248	30.66
		Error (133)	238,027.7		238,028	69.34
CBB (1–9 scale)	Year_1	RACE (1)	82.11	CLASS	0.29	5.87
		CLASS (8)	24.24 **	Error	1.25	25.6
		Error (201)	3.35		3.35	68.53
	Year_2	RACE (1)	72.14	CLASS	0.05	21.36
		CLASS (8)	29.75 **	Error	1.68	27.95
		Error (201)	1.78		1.78	50.68
	Year_3	RACE (1)	132.61	CLASS	0.51	8.1
		CLASS (8)	36.04 **	Error	1.93	30.41
		Error (201)	3.89		3.9	61.49
CR (1–9)	Year_3	RACE (1)	5.66 *	CLASS	0.04	30.77
		CLASS (6)	0.31 **	Error	0.01	7.69
		Error (266)	0.08		0.08	61.54

df = degrees of freedom; asterisks indicate * Significant at 5%, ** Significant at 1%.

**Table 3:** The analysis of variance for common bacterial blight and seed yield in common bean genotypes, races, and commercial seed classes from the Mesoamerican diversity panel evaluated in a greenhouse and in field experiments over three years.

Trait	Exp.	Variance Source (df)	Mean Square	Error	Variance Component	% of Total
		REP (2)	12.13	ERROR	0.09	5.17
Common Bacterial Blight (CBB)	Greenhouse	RACE (1)	4.25	CLASS	−0.12	0.00
		CLASS (RACE) (4)	16.92 **	GEN	0.31	17.82
		GEN(RACE*CLASS) (114)	2.44 **	ERROR	0.56	32.18
		Error (238)	0.08		0.78	44.83
	Field	YEAR (2)	25.69 **	ERROR	0.11	0.42
		RACE (1)	48.69	CLASS	0.53	13.23
		CLASS(RACE) (8)	65.56 **	GEN	0.27	11.61
		GEN (RACE*CLASS) (201)	8.36 **	ERROR	2.87	41.34
		ERROR (420)	1.43		1.43	33.40
Seed Yield per Hectare	Field	YEAR (2)	25,461,147.1 *	ERROR	119,825.7	21.1
		RACE (1)	5,899,456.6	CLASS	−12,323.3	0.00
		CLASS (RACE) (8)	5,983,223.5 **	GEN	66,726.9	11.75
		GEN (RACE*CLASS) (201)	710,453.7 **	ERROR	203,314.1	35.81
		ERROR (420)	177,933.9		177,933.9	31.34

df = degrees of freedom; asterisks indicate * Significant at 5%, ** Significant at 1%.

**Table 4:** List of significant Single-Nucleotide Polymorphism (SNP) markers associated with the CBB resistance disease score at the significance threshold cutoff value of *p* =1 × 10^−4^ (LOD = 4) in the common bean Mesoamerican diversity panel. Chromosomal physical position in base pairs (bp), association level (*p*-value), phenotypic variation explained by the SNP (coefficient of determination, R^2^), the positivity or negativity of the SNP effect, and the minor allele nucleotide identity with its corresponding Minimum Allele Frequency (MAF).

SNP Name	Chr.	Position	*p*-Value	R^2^	Effect	MAF
S10_1437174	10	1,437,174	1.7 × 10^−5^	0.034	−0.06	A (0.08)
S11_25981923	11	25,981,923	3.4 × 10^−5^	0.031	1.38	T (0.48)
S02_8541089	2	8,541,089	4.2 × 10^−5^	0.031	−0.35	G (0.49)
S08_24383486	8	24,383,486	4.4 × 10^−5^	0.031	−0.53	T (0.37)
S08_55730740	8	55,730,740	4.6 × 10^−5^	0.031	0.91	T (0.42)
S08_8738278	8	8,738,278	7.4 × 10^−5^	0.029	−2.55	A (0.50)
S04_29756136	4	29,756,136	7.9 × 10^−5^	0.029	0.85	A (0.25)
S05_23846983	5	23,846,983	8.3 × 10^−5^	0.029	1.39	G (0.20)
S05_25451953	5	25,451,953	8.3 × 10^−5^	0.029	0.83	G (0.10)
S11_25981955	11	25,981,955	8.4 × 10^−5^	0.029	−0.63	G (0.48)
S10_3398159	10	3,398,159	8.5 × 10^−5^	0.029	1.33	C (0.38)
S01_35716145	1	35,716,145	8.7 × 10^−5^	0.029	0.75	T (0.26)
S08_55730703	8	55,730,703	9.5 × 10^−5^	0.028	2.57	A (0.420
S11_25982002	11	25,982,002	9.7 × 10^−5^	0.028	−0.58	A (0.47)

## Data Availability

All genotypic data are publicly available. Phenotypic data available upon request and in files for the Supplemental Tables and Figure.

## Supplementary Materials

The following are available online at https://www.mdpi.com/article/10.3390/plants10050998/s1: Figure S1: Climate conditions for field experiments over three years in the state of Tennessee including daily precipitation, as well as maximum and minimum daily temperatures recorded over three growing seasons from April or May to August or September; along with the summary table of the weather data, Table S1: List of genotypes from the Mesoamerican common bean gene pool diversity panel used in this study, Table S2: One-degree-of-freedom orthogonal contrasts for CBB resistance scores comparing races, market classes within races, market classes between races, and CBB-resistant checks of 211 Mesoamerican DIVERSITY PANEL common bean genotypes grown over three years in Tennessee, Table S3: Mean yield performance and Standard Error of means (SE) for the top 5% of genotypes and bottom 2% of genotypes from the 211 entries of the Mesoamerican common bean diversity panel evaluated in the field over three years in Tennessee, Table S4: Mean disease score (based on the 1 to 9 scale) and SE of CBB on the top 13% of genotypes and the bottom 2% of genotypes from the 211 entries of the Mesoamerican common bean diversity panel evaluated in the field over three years in Tennessee, Table S5: The names, chromosomal positions, and functions of CBB candidate defense-associated genes identified in the genome-wide association study with the entries of the Mesoamerican common bean diversity panel in field and greenhouse testing in Tennessee.
