# Supplementary material for: Sources of Resistance to Common Bacterial Blight and Charcoal Rot Disease for the Production of Mesoamerican Common Beans in the Southern United States
Source: Plants (Basel). 2021 May 17;10(5):998. doi: 10.3390/plants10050998 (PMC8156677; doi:10.3390/plants10050998)
Supplement: Supplementary file 1 [file plants-10-00998-s001.zip › Suppl. Figure 1.pdf]

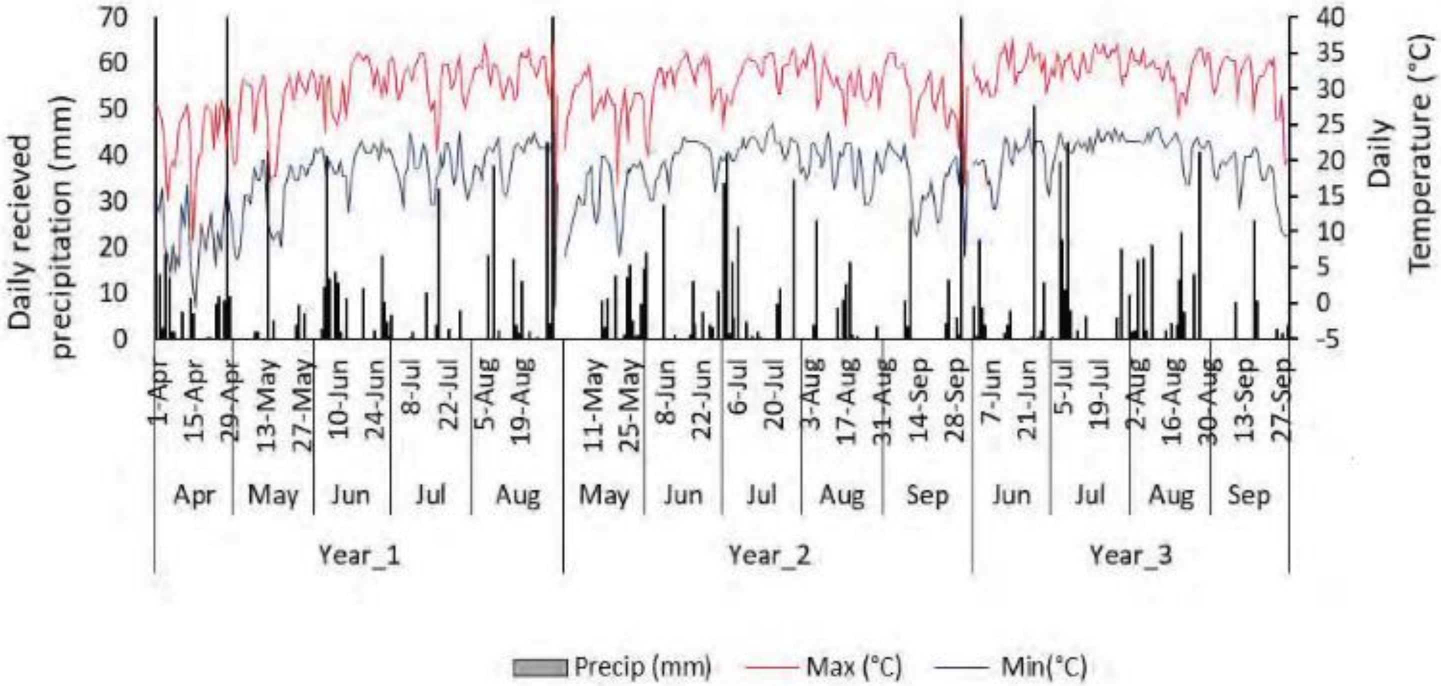

Weather Parameter	Year_1	Year_2	Year_3
Total Precipitation (mm)	592.82	489	483.63
Average Temperature (°C)	16.77-29.01	18.68 -30.39	20.65 - 32.53
Max Temperature Range (°C)	8.89-36.11	16.72 -36.11	19.39 - 36.72
Min Temperature Range(°C)	-0.5 -23.89	6.72 -25	9.39 - 24.39
